# Broad and Long-Lasting Vision Improvements in Youth With Infantile Nystagmus After Home Training With a Perceptual Learning App

**DOI:** 10.3389/fnins.2021.651205

**Published:** 2021-08-19

**Authors:** Bianca Huurneman, Jeroen Goossens

**Affiliations:** ^1^Department Cognitive Neuroscience, Donders Institute for Brain, Cognition and Behaviour, Radboud University Medical Centre, Nijmegen, Netherlands; ^2^Royal Dutch Visio, Nijmegen, Netherlands

**Keywords:** infantile nystagmus, perceptual learning, children, visual development, visual acuity, stereopsis

## Abstract

Current treatments for infantile nystagmus (IN), focused on dampening the oscillating eye movements, yield little to no improvement in visual functioning. It makes sense, however, to treat the visual impairments associated with IN with tailored sensory training. Recently, we developed such a training, targeting visual crowding as an important bottleneck in visual functioning with an eye-movement engaging letter discrimination task. This training improved visual performance of children with IN, but most children had not reached plateau performance after 10 supervised training sessions (3,500 trials). Here, we evaluate the effects of prolonged perceptual learning (14,000 trials) in 7-18-year-old children with IN and test the feasibility of tablet-based, at-home intervention. Results demonstrate that prolonged home-based perceptual training results in stable, long lasting visual acuity improvements at distance and near, with remarkably good transfer to reading and even stereopsis. Improvements on self-reported functional vision scores underline the clinical relevance of perceptual learning with e-health apps for individuals with IN.

## Introduction

Infantile nystagmus (IN) refers to bilateral, involuntary oscillating eye movements with an onset in the first 6 months of life. It is associated with suboptimal vision and affects the quality of life (for example driving difficulties, reliance on others and restricted career opportunities) (McLean et al., 2012). Clinical treatments for IN are missing, despite a significant prevalence of 1.4 per 1000 (Sarvananthan et al., 2009). Attempts to alleviate the life-long visual impairments by dampening the nystagmus have seen disappointing results. Acuity improvements (typically < 0.1 LogMAR) were much smaller than one might expect from the achieved attenuation of the nystagmus (McLean et al., 2007; Jayaramachandran et al., 2014), suggesting that one should (also) target bottlenecks in visual processing. A significant problem especially in individuals with IN is visual crowding, the inability to identify objects in clutter (Nandy and Tjan, 2012).

Interestingly, visual perceptual learning (VPL) – performance enhancement on a visual task as a result of visual experience (Sasaki et al., 2010) – proves an effective, non-invasive, personalized treatment strategy to improve visual functions (Deveau and Seitz, 2014). It is helpful in different patient populations such as subjects with amblyopia (Levi et al., 1997; Polat et al., 2004), macular degeneration [(Plank et al., 2014), also Stargardt disease (Sasso et al., 2019)], presbyopia (Polat et al., 2012), myopia (Tan and Fong, 2008; Camilleri et al., 2014a, b; Casco et al., 2014), and other forms of visual impairment (Huurneman et al., 2013, 2016a, b, c; Nyquist et al., 2016; Battaglini et al., 2021). Several recent studies indicate that VPL transfers to improvements in daily life visual functioning (Tan and Fong, 2008; Deveau and Seitz, 2014; Plank et al., 2014), underscoring the prospects of VPL as a valuable treatment option. The goal of the current study is to evaluate the effects of a home-based VPL app for youth with IN that targets visual crowding as an important bottleneck.

A frequently reported limitation of VPL is that learning effects are task-specific while effective therapies require generalization across visual tasks and stimuli (Polat, 2009; Deveau and Seitz, 2014). Successful ways to promote generalization are: (1) engagement of attention, use of (2) reinforcement techniques, and (3) stimuli with varying orientations, spatial frequencies, and locations (Deveau and Seitz, 2014). Our training app implemented these three approaches to foster generalization of learning by including an attentional demand (i.e., shifting visual attention), reinforcement techniques (by including feedback and performance-contingent rewards) and stimuli were offered in various orientations, spatial frequencies and locations. Another effective way to boost training effects is to increase the number of training sessions (Li et al., 2008, 2016; Polat, 2009). However, too much training may limit learning transfer (Jeter et al., 2010). Of note here is that VPL tends to occur in steps, with temporary plateaus of steady performance followed by further improvements before a subject finally reaches his or her best possible performance (Li et al., 2008).

Recently, we developed a perceptual training for children with IN which involved a two-step sequence in which participants were first presented with a central (probe) stimulus for 500 ms after which they had to redirect their attention and gaze toward a peripheral patch containing the uncrowded or crowded target letter as soon as it appeared. The children started their uncrowded or crowded training with stimuli that yielded 70% correct performance. During training, letter size (uncrowded training) or the letter size and letter spacing (crowded training) were modified according to the child’s training progress to maintain the same task difficulty level as children became progressively better at discriminating the letters. Children received trial-by-trial feedback about their performance in the form of a smiley, and were motivated with a reward game in between trial blocks. After ten training sessions of 350 trials each, both groups of children had improved vision even though their oculomotor behavior remained unaltered (Huurneman et al., 2016a). There was some task-specificity in the learning effects of the uncrowded versus crowded training with larger improvements of uncrowded acuity in the uncrowded training group and larger improvements in crowded acuity in the crowded training group (Huurneman et al., 2017), but there was considerable transfer to visual acuity charts, stereopsis and reading for both (Huurneman et al., 2016a, b, c).

However, a number of important questions are still outstanding. First, are these effects stable in the long term – a hallmark of perceptual learning? Based on VPL results in adults with amblyopia (Hussain et al., 2012) and our recent findings in children with low vision (Huurneman et al., 2020), one would expect an almost complete retention of visual acuity improvements and crowding reductions. Second, can a full contrast (high level) letter discrimination task result in improved contrast sensitivity (low level visual function) (Chung et al., 2012)? Most children had not reached plateau performance after 10 training sessions. Therefore, our third question is whether prolonged VPL yields larger training benefits? And if yes, are training benefits larger in observers with idiopathic IN than in observers with albinism and IN, as suggested by our previous work (Huurneman et al., 2017)? Last, but not least, can subjects take care of their own treatment? There are a few studies in which the feasibility of home-based VPL in subjects with amblyopia has been evaluated (Hussain et al., 2014; Hernandez-Rodriguez et al., 2021). These studies report compliance issues with home-training resulting in either too little training (e.g., 30-40% compliance, Hernandez-Rodriguez et al., 2021) or not complying to the recommended training duration (Hussain et al., 2014).

**FIGURE 1:**
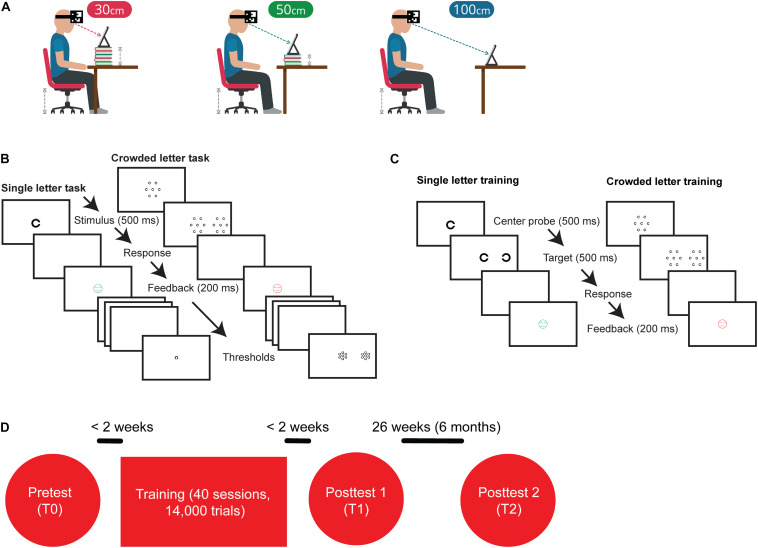
Training and experimental procedures. **(A)** Before the start of a training session, participants received visual and auditory instructions about the desired training configuration. Part of the training set-up was that the iPad’s camera tracked a QR-code attached to the subject’s head with a headband. During training, subjects gave responses by using a Nimbus game controller. **(B)** Outline of the single letter discrimination task. Subjects were instructed to indicate the orientation of the briefly (500 ms) presented C by pressing the corresponding arrow key on a keybord. Outline of the crowded letter discrimination task consisting of a two-step stimulus sequence. Subjects were instructed to fixate on the central patch, redirect their gaze toward the peripheral patch as soon as it appeared, and indicate the orientation of the C in the middle of that peripheral patch. Patches were only visible for 500 ms. **(C)** The single letter training contained the two-step procedure that was used in the crowded letter discrimination task, but did not present distractors surrounding the center probe and target. The crowded letter training was similar to the crowded letter discrimination task. **(D)** Flow chart of the experimental procedures. The complete training program consisted of 40 sessions of which the first 10 had an uncrowded letter configuration and the last 30 sessions had a crowded letter configuration.

To address these questions, the new training app enabled children to train at home without any direct supervision whereas the previous cohort of children practiced under supervision of a personal trainer. Viewing distance was monitored with the front camera of the tablet computer and the program gave audiovisual instructions concerning the required viewing distance (Figure 1A). The new training also combined uncrowded and crowded letter training (Figures 1B,C), and we increased the number of training sessions from 10 to 40 (Figure 1D).

We assessed task performance before and after the prolonged VPL training program and performed follow up measurements after 6 months. To quantify transfer of VPL, we measured improvements on visual acuity charts, changes in stereopsis, and reading performance. In addition, we assessed contrast sensitivity and we evaluated daily life visual function with a functional vision questionnaire (Tadic et al., 2013).

## Materials and Methods

### Participants

A total of 37 children and adolescents with IN were included. 19 children had albinism and 18 had idiopathic IN. Diagnostic groups were age-matched. The inclusion criteria were: age 7-18 years, diagnosis albinism in combination with IN or idiopathic IN, binocular crowded distance visual acuity (DVA) between 0.1 and 1.3 logMAR (VA ≥ 20/400 and <20/20), no additional impairments, born at term (>30 weeks) with normal birth weight (>3000 gr). Children were recruited either via Royal Dutch Visio (a Dutch vision rehabilitation center, *n* = 15) or via a patient network (“Nystagmus Netwerk Nederland,” *n* = 22). The diagnosis, clinical characteristics and refraction corrections of the participants are listed in Supplementary Table 1. Refractive corrections were checked within 12 months prior to inclusion and the children wore the same refraction correction throughout the study.

The study was conducted according to the principles of the Declaration of Helsinki and approved by the local ethics committee (CMO Arnhem-Nijmegen, protocol ID NL61860.019.17, Netherlands Trial Register, trial number NL6711). After explanation of the nature and possible consequences of the study, informed consent was obtained from the parents of all participants. Tests were performed at the Donders Institute for Brain, Cognition and Behavior.

### Apparatus

Stimuli for the pre- and post-training measurements were generated by a 15.6-inch laptop (Dell M4700) equipped with an open GL graphics card (NVIDIA Quadro K2000M) and presented on a 32-inch Liquid Crystal Display (LCD, Dell UP3214Q, 3840 × 2160 pixels; pixel pitch: 0.18 mm^2^). The training was executed on a 9.7 inch iPad Air 2 (2560 × 1536 pixels pixel pitch 264 ppi (0.077 mm^2^)). Background luminance of the 32-inch monitor, measured with a luminance meter (Minolta LS-100), was 0.3 cd/m^2^ for a black background and 193.8 cd/m^2^ for a white background. For the iPad Air 2, the luminance values were 0.9 cd/m^2^ for a black background and 409 cd/m^2^ for a white background. Experimental stimulus software for the pre- and post-training measurements was written in Matlab (version 2014b, MathWorks, Inc., Natick, MD, United States) using the Psychophysics Toolbox (version 3.0.12) (Brainard, 1997). Stimulus timing and button responses were recorded and stored at millisecond precision for offline analysis. Training software was written in Unity programming language.

### Procedure

**FIGURE 2:**
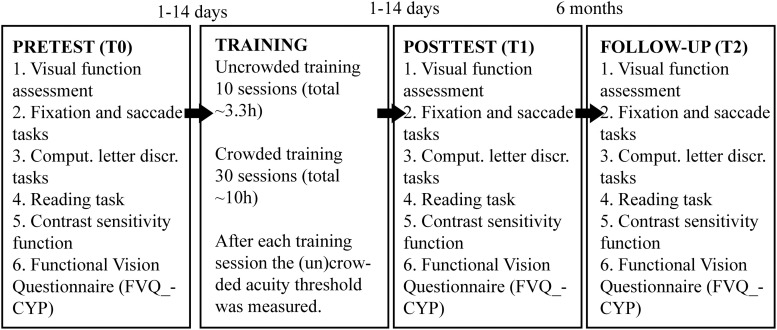
Schematic presentation of the experimental design.

The experimental procedure was the same for all participants. They underwent baseline measurements before training (T0) after which they trained twice a week for approximately 20 weeks. After training, the same measurements were collected as pre-training (T1). Six months later, a second set of post-training measurements (T2) were taken to determine long-term retention of training effects (see Figure 2). There was no explicit instruction with regards to supervision from parents. In principle, the interface was clear and children should be able to perform the task without supervision. However, there were some young children which did benefit from the supervision of an adult during training.

Pre- and post-training measures were administered in the following order: (1) a visual function assessment, (2) computerized letter discrimination tasks, (3) reading test, (4) contrast sensitivity measurement, and (5) the Functional Vision Questionnaire. A chin- and headrest was used to stabilize the head of our participants to keep them at the intended viewing distance.

### Pre/Post Training Measures

#### Visual Function Assessment

We assessed monocular and binocular DVAs, binocular near visual acuity (NVA) and stereopsis. DVA assessment was done with the commercially available ‘C-test’(Haase and Hohmann, 1982). DVA was measured binocularly at 5.0 meter with the uncrowded and crowded version of the ‘C-test’ acuity chart. A crowded chart version with an inter-letter spacing of 2.6 arc min and an uncrowded chart version with an inter-letter spacing of at least 30 arc min was used. This crowded chart was chosen because of its tight inter-letter spacing and its sensitivity to detect crowding effects (Neu and Sireteanu, 1997). Near visual acuity (NVA) was determined binocularly at 40 cm viewing distance with the LEA-version of the C-test (acuity range –0.3 to 1.7 logMAR, Huurneman et al., 2012). The Titmus Stereo Fly test was used for stereopsis measurements (Hasche et al., 2001). Finally, the crowding effect was calculated from the near and distance acuity measures by subtracting the uncrowded from the crowded logMAR acuity measures.

#### Computerized Letter Discrimination Tasks

Two computer-controlled letter discrimination tasks were used to determine uncrowded letter VA, crowded letter VA and crowding extent under a 500 ms time constraint. Letters were high-contrast (99.7% Michelson) black Landolt Cs (0.3 cd/m^2^) with 4 possible orientations against a white background (193.8 cd/m^2^). Both four alternatives forced choice letter discrimination tasks consisted of two phases: (1) a short two up one down staircase procedure to get an initial estimate of the acuity of the subject (40 trials), and (2) a more extensive constant stimuli procedure in which Landolt C rings of six different sizes were each presented 15 times in pseudorandom order.

During the single letter discrimination task, a central C was presented for 500 ms in one of four orientations. Subjects reported the orientation of the C by pressing the corresponding arrow key on a keyboard and then received feedback about the correctness of their answer by presenting a green (smiling) or red (neutral) smiley for 200 ms (Figure 1B) for correct and incorrect answers, respectively. To obtain an initial estimate of the subject’s letter discrimination threshold, a short staircase procedure was adopted. The first stimulus of the staircase was always 1.3 logMAR. Step size was initially 0.2 logMAR, but this was lowered to 0.1 logMAR after two reversals. The threshold was calculated from the mean of the last six size reversals. Subsequently, discrimination performance was evaluated with the constant stimulus procedure in which six letter sizes were presented in pseudorandom order. Letter sizes ranged from 0.2 log units below to 0.3 log units above the threshold estimated with the staircase procedure. For each letter size, 15 trials were presented, leading to a grand total of 90 trials. A Weibull function was fitted to the resulting psychometric response function to find the 62.5% correct discrimination threshold.

The crowded letter discrimination task consisted of a two-step stimulus sequence. Trials started with a central stimulus probe consisting of a group of 7 Landolt C with the gaps on the same side. After 500 ms, a second stimulus appeared to the left or to the right of the probe. The second stimulus consisted of a target C surrounded by 6 flanking Cs of the same size in another orientation. The target C had a different orientation than the surrounding Cs, which were placed at 60 deg intervals around the target (Figure 1C). Children were instructed to identify the orientation of the target C by pressing the corresponding keyboard button. As was the case for the single letter discrimination task, trial-by-trial feedback was given with a smiley. For children with crowded DVA < 0.7 logMAR eye-to-screen distance was 150 cm and eccentricity of the crowding stimulus was 5 degrees to prevent the second patch from falling outside the screen. For children with crowded DVA ≥ 0.7 logMAR eye-to-screen distance was 50 cm and crowded patch eccentricity was 15 degrees to prevent patches from overlapping. The minimum letter size needed to perform the task was first estimated with a staircase procedure. The letter sizes on the first trial were set equal to the single-letter discrimination threshold. The initial step size was 0.2 logMAR and after two reversals, the step size was reduced to 0.1 logMAR. Target to flanker distance was relative to letter size (center-to-center spacing as a multiplication of letter height was × 3). The discrimination threshold was estimated from the mean of the letter size of the last six reversals. Subsequently, the letter spacing threshold was determined with a set of constant stimuli. In this procedure, letter sizes were fixed at the threshold determined from the crowded letter staircase procedure with a 0.1 logMAR enlargement. This small increase in letter size was used to make sure that the psychometric response function for the spacing threshold also covered the 70-100% correct range. The following six center-to-center spacings were presented in pseudorandom order (expressed as a factor of stimulus height): ×1.2, ×1.5, ×2.0, ×2.5, ×3, and ×4. The 62.5% correct discrimination thresholds were determined from Weibull function fitted to the psychometric response curve. The spacing thresholds were expressed in logMAR: crowding extent = log_10_(crowded letter threshold × ‘critical’ center-to-center spacing factor/5) where crowded letter threshold is the crowded letter acuity expressed in minutes of arc visual angle and ‘critical’ center-to-center spacing factor the letter spacing factor corresponding to the 62.5% correct discrimination threshold.

#### Reading

Reading performance was assessed with a computerized version of the Radner reading test (Maaijwee et al., 2008). Sentences were composed of high-contrast black letters (0.3 cd/m^2^) presented on a white background (193.8 cd/m^2^; 99.7% Michelson contrast). Sentences were presented in Arial font type. Before sentences were presented, crowded DVA was entered as an initial estimation of reading acuity. Eight sentences from the Dutch reading chart LEOntientje were presented to determine the minimum font size that could be read correctly without making errors (font size range –0.2:0.1:0.5 logMAR). After determining reading acuity, children were instructed to read 24 sentences containing 14 words out loud as fast and accurately as they possible. Font size ranged from 0 to 0.75 logMAR above the children’s reading acuity with steps of 0.15 logMAR (4 sentences per font size × 6 font sizes = 24 trials). Reading distance was 150 cm for children with crowded DVA of ≤0.3 logMAR, 50 cm for children with crowded DVA between 0.4 and 0.8 logMAR, and 20 cm for children with crowded DVA ≥ 0.9 logMAR. The shorter viewing distance for children with poorer acuities was adopted because the number of words on a line was restricted by the screen dimensions and font size.

The following outcome measures were extracted from the reading data: (1) reading acuity (RA), i.e., the smallest font size (in logMAR) that children could read without making errors, (2) maximum reading speed (MRS) determined from the mean of the three fastest reading speeds, (3) critical print size (CPS), the smallest font size in logMAR that could be read at 80% of the maximum reading speed estimated by linear interpolation between data points, and (4) acuity reserve (AR), the enlargement above the RA needed to reach 80% of the maximum reading speed, calculated by subtracting the RA from the CPS.

#### Contrast Sensitivity Function

Contrast sensitivity was measured with a customized procedure in which a black Landolt C stimulus was presented at 150 cm on a gray background and luminance of the stimulus letter was adjusted. Children were informed about the correctness of their answer with auditory feedback. Screen luminance was calibrated prior to the experiments. Clinically available contrast chart like the CSV-1000 typically consists of 5 spatial frequencies (3, 6, 12, and 18 cycles per degree or 1.0, 0.7, 0.4, and 0.2 logMAR). But, since the crowded DVAs vary considerably between subjects with IN, these charts do not allow measuring a wide range of contrast sensitivities within the appropriate range of spatial frequencies for all subjects. For subjects with DVA ≤ 0.2 logMAR, the following letter sizes were presented: 1.3, 1.0, 0.7, 0.4, and 0.2 logMAR. If DVA was larger than 0.2 logMAR, then the range of letter sizes was shifted so that the smallest presented letter was equal to their crowded DVA (smallest identifiable letter size at full contrast). An adaptive staircase procedure (Quest, Watson and Pelli, 1983) was used to determine the 62.5% correct contrast threshold for the each of the 5 letter sizes. There were 30 trials for each letter size, leading to a grand total of 150 trials. Weber contrast (i.e., luminance of the letter minus the luminance of the background divided by the luminance of the background) was used to define contrast thresholds. Contrast thresholds were expressed in log contrast thresholds, e.g., –1.50 log units equals a contrast threshold of 3% and a contrast sensitivity of 33 (1/0.03).

#### Functional Vision Questionnaire

A validated questionnaire containing 36 items was used to evaluate the impact of vision loss on a person’s daily life. The questionnaire, The Functional Vision Questionnaire for Children and Young People (FVQ_CYP) (Tadic et al., 2013, Elsman et al., 2019), comprises items that are organized into four categories of activity: home (8 items), school (16 items), sports (7 items), and leisure (5 items). The questionnaire contains items on which children can answer on a 5-point scale to rate their level of functioning from “very easy” (score 0), “easy” (score 1), “a little bit difficult” (score 2), and “very difficult/impossible” (score 3). There was also an answer option “this doesn’t apply to me.” Sample activities include seeing the board in class, playing sports, getting around school unassisted, using escalators, reading price tags, and finding the correct money to pay for items. The FVQ captures a child’s personal perspective on his or her daily visual functioning, particularly over time and can be used to evaluate the effectiveness of rehabilitation or interventions.

### Training Task

All subjects trained at home with an iPad Air 2 (for specifications, see section ‘Apparatus’). Before subjects started their home training, they received verbal instructions on the day of the pre training measurement on how to set up the tablet computer (an iPad), how to wear the QR-code on their forehead for monitoring actual viewing distance, and how to use the response buttons on the game controller. They also received a written training protocol with instructions concerning the desired training frequency, technical instructions on how to set up the iPad at home and a phone number that they could call in case they needed help. At the end of each training session, the app sent log data of that session to a server, allowing us to monitor training compliance remotely.

In addition, pre training letter discrimination thresholds were entered as a starting point for the training. The training consisted of 10 uncrowded training sessions followed by 30 crowded training sessions. Because foveal vision is generally limited by three factors – uncrowded visual acuity, crowding and overlap masking (Song et al., 2014) – we hypothesized that stacking the uncrowded and crowded training might enhance the net learning. We chose to start with 10 uncrowded training sessions followed by 30 crowded training sessions based on the observation that uncrowded letter improvements occur over a shorter time course than crowded letter improvements (Li et al., 2007; Huurneman et al., 2016c). We reasoned that by improving the uncrowded acuity first, the effect of the crowed training might be greater if uncrowded visual acuity is one of the bottlenecks for improving crowded visual acuity. Both the uncrowded and crowded training sessions involved a two-step stimulus sequence where subjects are first presented with a central probe for 500 ms, after which the target is presented on the left or right sight of the screen. The objectives of implementing this two-step routine were to train oculomotor- and attention control, and possibly invoke more generalized learning.

In the uncrowded training task, both the central probe and the eccentric target stimulus were Landolt C rings presented at the subject’s single letter discrimination threshold found during pretest. During the crowded training sessions, the central stimulus patch consisted of a central Landolt C letter surrounded by 6 Landolt C letters (with the same orientation). The eccentric target was a Landolt C (with a different orientation) surrounded by 6 distractors. Subjects started their first uncrowded training at the letter size found at the pre training measurement. During the uncrowded training sessions, stimulus size was reduced with 0.1 logMAR if at least 7/10 answers were correct. During the crowded training sessions, subjects started their first crowded training session with the letter size and spacing factor determined at pretest. Letter spacing was reduced by 0.1 log units if at least 7/10 answers were correct, if less than 7 out of 10 answers were correct spacing was increased by 0.1 log units. If letters were closer to each other than 1.1 × the letter size, letter size was reduced by 0.1 log units and the target-to-flanker spacing was adjusted to 3 × the letter size. At the end of each training session a 2-up-1-down staircase consisting of 40 trials was used to determine either the discrimination threshold for single Landolt Cs (after an uncrowded training session) or the discrimination threshold for crowded Landolt Cs (after a crowded training session, same configuration as the crowded letter stimulus shown during training).

Each training session consisted of 7 blocks of 50 trials. In between blocks, children played a reward game Huurneman et al. (2016c). Children were asked to train 2 × per week for 20 consecutive weeks, making a grand total of 14,000 trials per child. Subsequent training sessions always started at the letter discrimination threshold measured with the staircase procedure at the end of the previous training session.

Training sessions opened with a screen providing the subjects information about the number of completed training sessions and the viewing distance that should be adopted. The following viewing distances were possible: 30 cm, 50 cm, 100 cm, or 150 cm. Children responded to stimuli on the iPad by using a Nimbus game controller.

Actual viewing distance was monitored by tracking a calibrated QR code that the subject wore during training with the iPad’s built-in camera (Figure 1A). Subjects received a warning whenever the QR code was not visible, or when the viewing distance was >10% larger or smaller than the intended viewing distance. The training app gave both visual as well as auditory instructions to facilitate our participants. After each training session, the app sent a pseudo-anonymized, encrypted file containing all training log information to our secure network server. This way compliance to training rules could be monitored remotely (e.g., training frequency, visibility of the head target and compliance to instructions regarding viewing distance). In case anomalies were noticed for more than two or three sessions in a row, our help-desk contacted the child’s parents to determine the possible cause and provide assistance as needed.

### Data Analysis

Data were analyzed in MATLAB R2014b (Mathworks, Inc., Natick, MD, United States). Baseline performance measures were compared for children with albinism and children with idiopathic IN using an independent samples *t*-test.

Short-term training effects were evaluated with 2-way Repeated Measures ANOVAs. Sphericity assumptions were checked with Mauchly’s test. In case sphericity assumptions were not met, adjustments have been made and Greenhouse-Geisser corrected *p*-values were reported. Diagnosis, age and number of completed training sessions were entered as between subjects factors and pre- and post-test as a within subjects factor (T0 and T1). Unless mentioned otherwise, there was no effect of the between subject factors. Outliers (defined as scores outside ± 3 SD range from mean score) regarding training outcome were assigned a new value by imputation (i.e., using the mean difference score; *n* ≤ 2 per measure). In case training effects were diagnosis-dependent, a *post hoc* analysis was performed for the two diagnostic groups separately. For our contrast sensitivity measure, there were five levels for the pre- and post-test measurements.

Long-term training effects were determined with 3-way Repeated Measures ANOVAs with again diagnosis, age and number of training sessions as between subjects factors and pre- (T0), post1 (post-test directly after training, T1), and post2 (follow-up at 6 months after training, T2) as within subject factors. Results were considered statistically significant if alpha (Type I error) was <0.05. A Bonferroni correction was applied for *post hoc* tests and multiple comparisons.

A three parameter two line segment fit (Li et al., 2008) was used to model data collected during crowded training (last letter size presented during training). In formula:

Crowdedletteracuity(TS)=β0+(β1×TS)+(β2×TS),

where β0 stands for the baseline crowded letter acuity, β1 reflects the slope of the first line, and β2 reflects the slope of the second line segment (which was always 0). TS is short for training session. Our model predicted which fit was the best by iterating the formula for training session until the last training session that was performed by the subject (this number varied as can be seen in Supplementary Table 1). At the end we evaluated at which training session the root mean square value had the smallest value. If a linear fit was better than all the two segment models, then there was no apparent plateau to be found in the data and the last training session would provide the best fit.

## Results

One 7-year old boy dropped out after 9 training sessions due to a lack of motivation. Unfortunately, he did not show up for a post-training measurement and could therefore not be included in the analyses. All other participants (*n* = 36) underwent baseline measurements before training (T0) and then trained twice a week for approximately 20 weeks (for details concerning child characteristics and number of completed training sessions, see Supplementary Table 1).

### Baseline Differences Between Age-Matched Diagnostic Groups

On average, children with albinism (all having IN, *n* = 19 of which 11 had oculocutaneous albinism and 8 had ocular albinism) had poorer baseline binocular crowded distance visual acuities and stereopsis than children with idiopathic IN (IIN, *n* = 17) (Supplementary Table 2). Binocular crowded DVA’s were 0.71 ± 0.23 logMAR (mean ± SD) in children with albinism and 0.52 ± 0.14 logMAR in children with IIN. Stereopsis was 2.71 ± 0.48 log10 arc sec in children with albinism and 2.37 ± 0.51 in children with IIN. However, the crowding intensity (CI), expressed as the difference (in logMAR) between crowded and uncrowded visual acuity (Huurneman et al., 2016c), was not significantly different between these two diagnostic groups.

### Home Training

The log data showed that compliance with the training protocol was good. The QR-code on the subjects’ forehead was visible 88% of the training time (95% confidence interval 72–100%). The average gain factor of recorded viewing distance relative to instructed viewing distance (30, 50, 100, or 150 cm, depending on visual acuity) was 0.96 (95% confidence interval 0.92–1.00), indicating that participants typically adopted a viewing distance that was ∼4% closer than instructed. This value lies within the 10% distance limit implemented in the app. Larger distance deviations triggered a warning message on the screen. Supplementary Table 1 lists the number of completed training sessions for all participants. The majority, 25 subjects (69%), completed all 40 training sessions.

**FIGURE 3:**
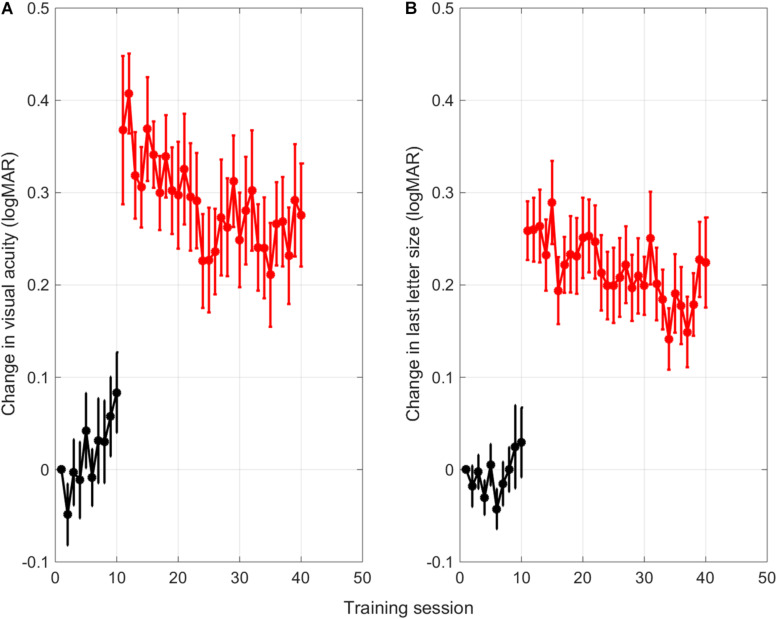
Averaged learning curves. **(A)** Change in uncrowded (black) and crowded (red) letter discrimination thresholds with respect to the single letter discrimination thresholds measured at baseline. Letter discrimination thresholds were measured after each uncrowded (session 1-10) and crowded (session 11-40) training session with a short staircase procedure (Methods). **(B)** Change in size of the last-presented letter of a training session across the different training sessions. Error bars: ±1 s.e.m.

As can be seen in Figure 3, training resulted in a gradual improvement of crowded acuity. Figure 3A shows the learning curve as a change in uncrowded (black) and crowded visual acuity (red) as determined with a 40-trial staircase procedure at the end of each uncrowded training (session 1–10) or crowded training (session 11–40), respectively. Figure 3B shows the training progress as measured from the size of last letter presented in that training session. All data are adjusted for single letter visual acuity at baseline to allow for averaging across subjects. The sudden increase in acuity values from training 11 onward therefore reflects the difference between crowded and single letter discrimination thresholds.

Surprisingly, the thresholds for uncrowded letter discrimination (black) seemed to go up over the first 10 training sessions with single letters. Linear trend analysis showed that this was the case for thresholds found with the single letter task that was performed after each training (*r* = 0.82, *p* = 0.003). However, for the size of the last letter that was presented in each training session, the trend was not significant (*r* = 0.47, *p* = 0.173). The discrepancy between the two measures might be due to the fact that the visual acuity measurements with the staircase procedures were done after the children just completed an entire training session of 350 trials.

Regression analyses performed on the following crowded training sessions (red learning curves, session 11-40), confirmed that the crowded letter discrimination improved significantly during training for both the threshold data and the size of the last letter shown in each session (threshold data Figure 3A: *r* = –0.70, *p* < 0.001; last letter size Figure 3B: *r* = –0.66, *p* < 0.001).

### Improved Task Performance After Training

Thirty-six subjects came for post-training measurements.

Two computerized tests were administered before and after training, i.e., a single letter task and a crowded letter task (Figure 1C). Pre-training results were used to determine starting levels of the uncrowded and crowed training, respectively. The configuration of the crowded letter task was identical to the configuration of the crowded letter training. The single letter task was not completely similar to the uncrowded letter training, because it did not include the stimulus jump.

#### Single Letter Acuity

**FIGURE 4:**
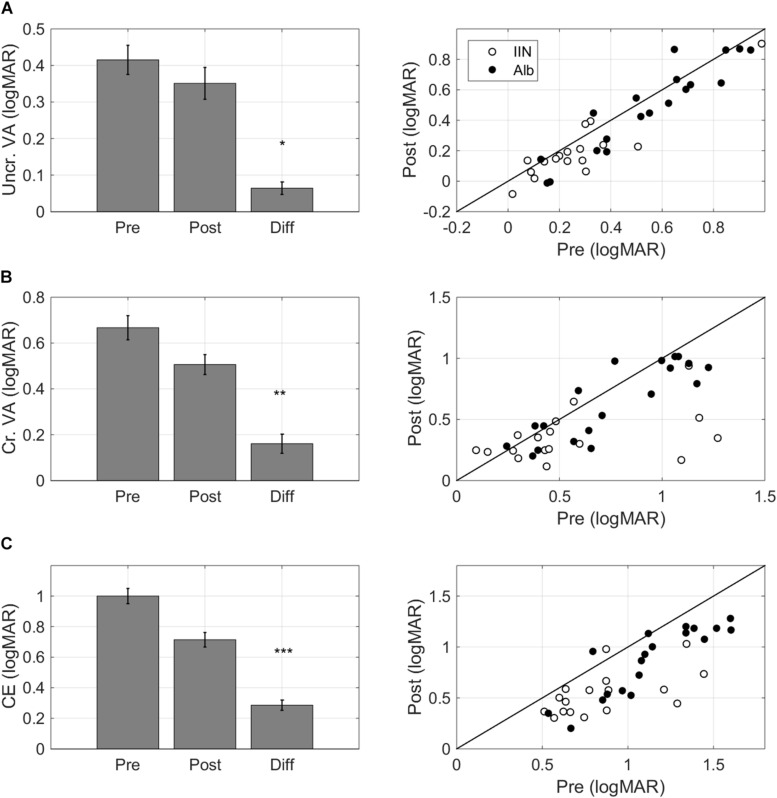
Task-specific changes after training. Pre training (Pre), post training (Post), and Difference (Pre training minus Post training) scores for **(A)** single letter acuity as measured with the single letter discrimination task, **(B)** crowded letter acuity as measured with the crowded letter discrimination task, and **(C)** crowding extent as measured with the crowded letter discrimination task. Positive difference values indicate improvements. The left-hand panels present bar graphs with data pooled for the two diagnostic groups. The right-hand panels present scatter plots where pre training data is presented on the horizontal axis and post training data is presented on the vertical axis. Data are split by diagnosis Albinism (Alb) and idiopathic IN (IIN). Points below the identity line indicate improvements. Error bars: ±1 s.e.m. *p < 0.05, **p < 0.01, and ***p < 0.001.

Training resulted in improvements in single letter acuity as measured by the single letter task (*F*(1,32) = 7.13, *p* = 0.012, ΔT0-T1 0.064 ± 0.017 logMAR, mean ± standard error of the mean, medium effect size Cohen’s d 0.62, Figure 4A).

#### Crowded Acuity

Crowded acuity as measured with the crowded letter task also improved with training (*F*(1,32) = 9.49, *p* = 0.004, ΔT0-T1 0.16 ± 0.04 logMAR, medium effect size Cohen’s d 0.64, Figure 4B).

#### Crowding Extent

The crowding extent, i.e., the minimum distance needed between a threshold-size target and flankers to allow target recognition in the crowded letter task (Huurneman et al., 2012), was reduced after training (*F*(1,32) = 37.55, *p* < 0.001, ΔT0-T1 0.29 ± 0.03 logMAR, large effect Cohen’s d 1.42, Figure 4C).

### Learning Transfer – Visual Acuity and Stereopsis

#### Distance Visual Acuity

**FIGURE 5:**
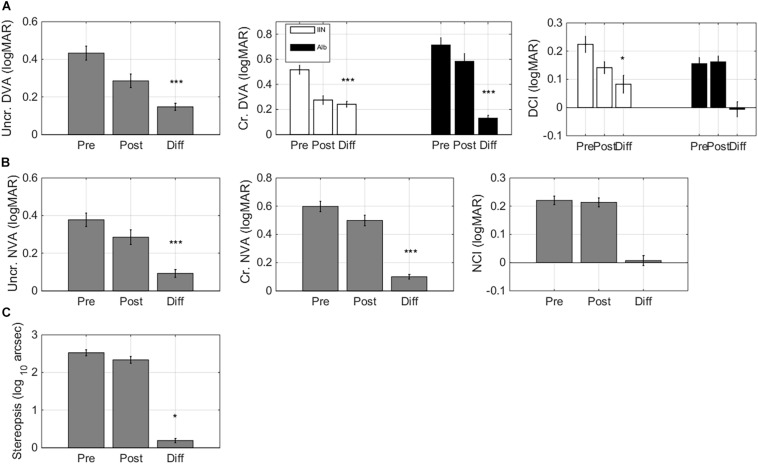
Learning transfer – visual acuity andstereopsis changes. Pre training (Pre), post training (Post), and Difference (Pre training minus Post training) scores for: **(A)** uncrowded and crowded distance visual acuity (DVA) and crowding intensity (DCI). Crowded DVA and DCI are split by diagnosis Albinism (Alb) and idiopathic IN (IIN). **(B)** Uncrowded and crowded near visual acuity (NVA) and crowding intensity (NCI). **(C)** Stereopsis. Error bars: ±1 s.e.m. *p < 0.05, ***p < 0.001.

Training resulted in uncrowded DVA improvements (*F*(1,32) = 31.11, *p* < 0.001, ΔT0-T1 0.15 ± 0.02 logMAR, Cohen’s d 1.30, Figure 5A).

Improvements in crowded DVA were diagnosis-dependent (*F*(1,32) = 9.46, *p* = 0.004, Figure 5A). Children with idiopathic IN showed large improvements (ΔT0-T1: 0.24 ± 0.02 logMAR, *F*(1,14) = 112.67, *p* < 0.001, Cohen’s d 2.57). Children with albinism also showed significant, but smaller crowded DVA improvements (ΔT0-T1 0.13 ± 0.02 logMAR, *F*(1,16) = 35.28, *p* < 0.001, Cohen’s d 1.36).

Changes in distance crowding intensity (DCI) were also diagnosis-dependent (Figure 4B): children with IIN showed a reduction in their DCI (ΔT0-T1 0.08 ± 0.03 logMAR, *F*(1,14) = 6.89, *p* = 0.020, Cohen’s d 0.64) while children with albinism showed no significant change (*p* > 0.8).

#### Near Visual Acuity

Both uncrowded NVA and crowded NVA were improved after training (*F*(1,32) = 13.82, *p* < 0.001 and *F*(1,32) = 20.09, *p* < 0.001, respectively. Δ pre-post 0.09 ± 0.02 logMAR and 0.10 ± 0.02 logMAR, Cohen’s d 0.74 and 1.02). Near crowding intensity did not change signficantly (*F*(1,32) < 1).

#### Stereopsis

Even though the training did not include depth perception, it resulted in significant stereopsis improvements (*F*(1,32) = 4.91, *p* = 0.034; ΔT0-T1 0.19 ± 0.06 log(10) arc sec, Cohen’s d 0.57, Figure 5C).

### Learning Transfer – Reading Performance

To determine reading curves, reading speed was measured for six font sizes (ranging from smallest readable font size with increasing steps of 0.15 logMAR). One boy was too young to perform the reading test at T0 (ID 2) and was therefore excluded from the reading analysis (total data set, *n* = 35).

**FIGURE 6:**
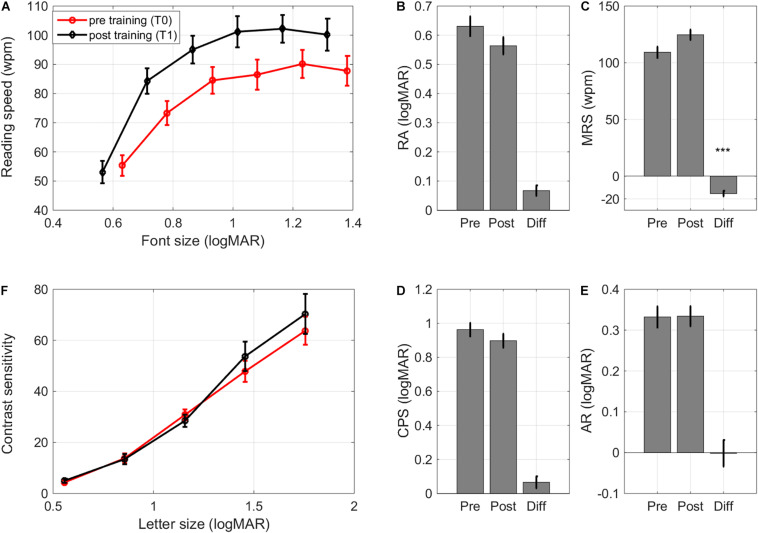
Learning transfer – reading and contrastcurves. **(A)** Pre and post training reading curves averagedacross subjects, **(B)** changes in reading acuity, **(C)** changes in maximum reading speed, **(D)** changes in critical print size, **(E)** changes in acuity reserve, and **(F)** pre and post contrast sensitivity curves (contrast sensitivity of 100 indicates a contrast sensitivity threshold of 1%, i.e., contrast sensitivity/100 = % Weber contrast). Error bars: ±1 s.e.m. ***p < 0.001.

After training, there were no significant improvements in reading acuity (RA, i.e., the size of the smallest readable font. Figures 6A,B, *F*(1,31) = 3.87, *p* = 0.058, Cohen’s d 0.62). Maximum reading speed (MRS) improved significantly after training (*F*(1,31) = 14.41, *p* < 0.001, Δ pre-post = –15 ± 2 wpm, Cohen’s d 1.04, Figure 6C). Age affected the changes in reading speed (*F*(1,31) = 4.55, *p* = 0.041): younger children showed larger improvements in maximum reading speed (*r* = 0.36). Since negative pre-post differences in MRS indicate improvements in reading speed, the positive correlation indicates that older children tended to show smaller improvements in reading speed.

Critical print size (CPS), the smallest font size with which children could achieve at least 80% of their MRS, and acuity reserve (AR), the difference between the smallest readable print size and CPS, were unaltered (*F*(1,31) < 1, Cohen’s d 0.31 and 0.01, respectively. Figures 6D,E). Training effects on reading performance were similar for children with idiopathic IN and children with albinism, even though the latter group had poorer RA and CPS at baseline (Supplementary Table 3).

### Learning Transfer – Contrast Sensitivity

There is evidence that contrast sensitivity for small letters can improve after perceptual learning (Polat, 2009), even if the training paradigm only includes high (100%) contrast crowded letters (Chung et al., 2012). Contrast sensitivity was therefore determined for a range of letter sizes. For subjects with a crowded DVA ≤ 0.2 logMAR the sizes of presented letters were 1.3, 1.0, 0.7, 0.4, and 0.2 logMAR. If the crowded DVA was weaker than 0.2 logMAR, the range of letter sizes was shifted with the smallest letter size corresponding to the subject’s crowded DVA. Contrast sensitivity measurements were collected for 23 children (see Supplementary Table 3 for baseline data).

As expected, contrast sensitivity depended significantly on letter size (*F*(4,84) = 55.99, *p* < 0.001, Figure 6F). Neither diagnosis nor training affected the contrast sensitivity curve (diagnosis: *F*(1,21) < 1;training: *F*(1,21) < 1; training × letter size interaction: *F*(4,84) < 1. Figure 6F). There were no differences in changes between diagnostic groups either (*F*(1,21) < 1).

### Self-Reported Improvements in Functional Vision

#### Functional Vision Questionnaire

**FIGURE 7:**
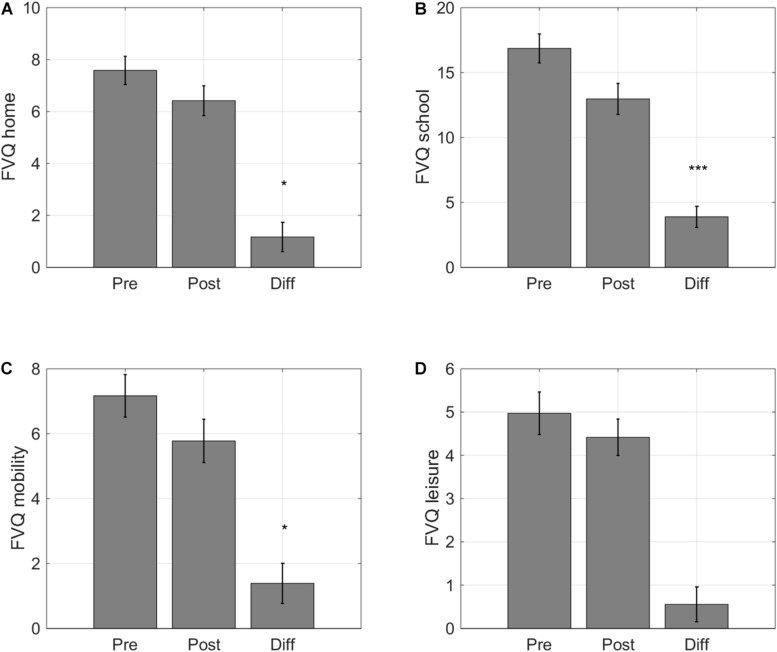
Self-reported changes in functional vision. Average scores for the four activity categories of the Functional Vision Questionnaire for Children and Young People (FVQ_CYP): **(A)** home, **(B)** school, **(C)** sports, and **(D)** leisure. Lower VFQ scores indicate that childrenexperience a smaller impact of their visual impairment on daily lifevisual tasks. Error bars: ±1 s.e.m. *p < 0.05 and ***p < 0.001.

Previous VPL studies have assessed visual functioning through psychophysical tests, as we have done above, but have, to our knowledge, not addressed how training influences daily life activities in individuals with IN. We found that training did ameliorate the impact of the subjects’ visual impairment on their daily life activities. Scores improved on three of the four subscales. Participants gave a lower score on the subscale ‘Home’ after training (*F*(1,32) = 4.17, *p* = 0.049, Cohen’s d 0.34, Figure 7A), indicating that they could perform activities at home with greater ease (e.g., watching television, doing household chores, or telling the time on a wall clock). There was a large impact on the ease with which participants felt they could perform their school activities (*F*(1,32) = 20.00, *p* < 0.001, Cohen’s d 0.80, Figure 7B). The scores also indicted a positive impact of training on mobility activities (*F*(1,32) = 5.06, *p* = 0.031, Cohen’s d 0.37, Figure 7C). However, children did not report changes in their visual impairment on leisure activities (*F*(1,32) = 2.18, *p* = 0.150, Cohen’s d 0.23, Figure 7D).

### Long-Term Retention of Training Effects

Follow-up measurements were collected 6 months after training in 32/36 participants. One of these 32 children did not complete the computer measurements at T2 (ID 6). Long-term retention of training effects was evaluated in these participants by conducting a 3-way Repeated Measures ANOVA (T0-T1-T2) with the same between subjects factors entered as for the 2-way Repeated Measures ANOVAs. We only considered outcome measures for which we found significant short-term training effects.

**FIGURE 8:**
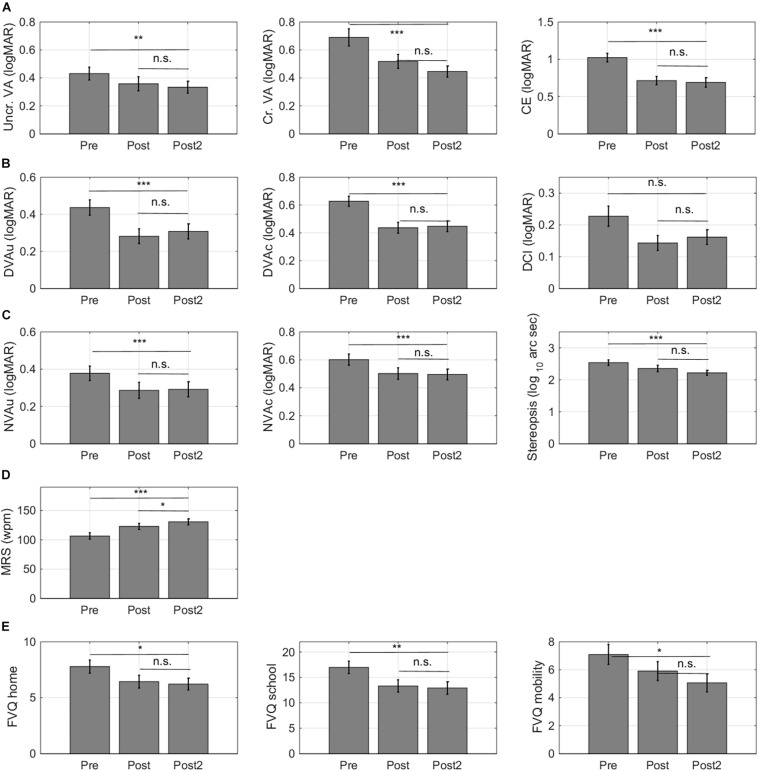
Retention of learning effects. For 32/36 subjects, follow-up measurements were collected 6 months after training. **(A)** Training effects measured with the computerized letter discrimination tasks. **(B)** Training effects measured with the distance visual acuity (DVA) charts. **(C)** Training effects measured with near visual acuity charts (NVA). **(D)** Training effects on reading speed. **(E)** Training effects on self-report measures of functional vision. Lower VFQ scores indicate that children experience a smaller impact of their visual impairment on daily life visual tasks. Error bars: ±1 s.e.m. *p < 0.05, **p < 0.01, ***p < 0.001, and n.s., not significant.

As shown in Figure 8, the improvements remained stable over time for 12/13 outcome measures. The small impact of training on DCI in children with idiopathic IN was not significant at 6 months after training. Maximum reading speed (MRS) showed a further increase. For the computerized letter discrimination tasks, the difference between the pre-training and the follow up measures taken at 6 months after training (ΔT0-T2) was still highly significant (*p*’s < 0.010), while there were no differences between the post-test and 6 month follow-up measurements (ΔT1-T2) (Figure 8A).

Six months after training, improvements in uncrowded and crowded distance (Figure 8B) and near visual acuity (Figure 8C) were completely intact. Maximum reading speed showed further improvement between the first and second post-test measure (Figure 8D). Notice that this improvement was two times smaller (ΔT1-T2 –8 ± 3 wpm) than the improvement that occurred between the pretest and the first post-test (ΔT0-T1 –16 ± 3 wpm). Finally, improvements on the functional vision questionnaire also remained intact over time (Figure 8E).

### Number of Sessions Needed to Reach Plateau Performance

**FIGURE 9:**
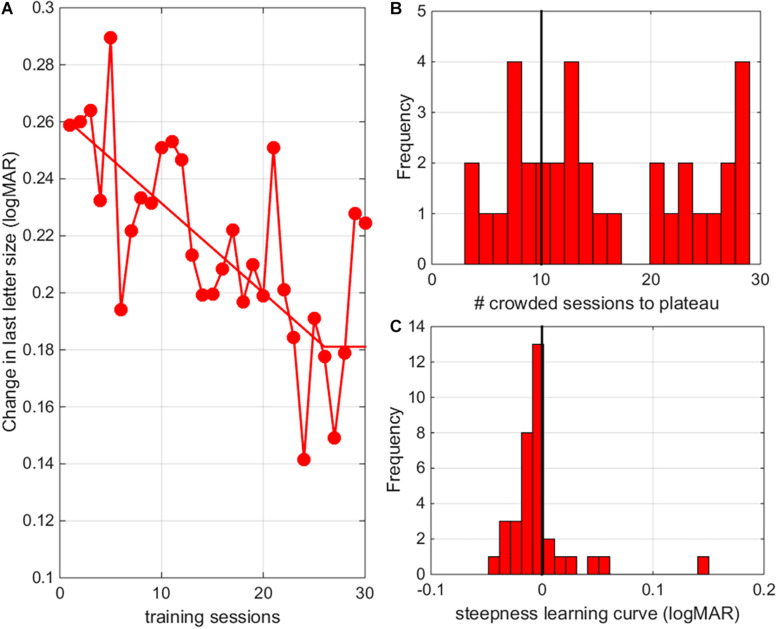
Number of sessions needed to reach plateau. **(A)** The average change in crowded letter acuity as a function of training session (results pooled for all individuals). Responses were fitted with a three-parameter two-line segment curve to determine performance thresholds as a function of training session with the intersection point of the two lines indicating plateau performance. **(B)** Histogram of the number of training sessions needed to reach plateau performance. **(C)** Histogram of the steepness of learning curves.

Following previous approaches, we used a three-parameter two-line segment curve to fit performance changes as a function of training session with the intersection point of the two lines indicating plateau performance (Li et al., 2008). When looking at the average learning curves, children reached plateau performance after 26 crowded training sessions (Figure 9A, for individual learning curves see Supplementary Figure 1). However, there was a lot of variability between subjects in the steepness of learning curve and in the number of training sessions needed to reach a stable learning plateau (Figures 9B,C). The number of training sessions needed to reach plateau performance and steepness of the learning curves could not be predicted by patient characteristics (i.e., diagnosis, age or baseline performance, *r*’s < 0.2). The total reduction of the acuity threshold determined from the training data, however, was correlated with the initial acuity values (*r* = –0.37, *p* = 0.029).

## Discussion

Our study indicates that children and adolescents with IN can use an e-health solution to train their visual functions. This is an important step toward more patient independence. The training program not only improves performance on the trained letter-discrimination tasks, but also improves visual acuities measured with clinical vision charts. Importantly, we also observed broad transfer of training effects to untrained tasks such as reading performance, untrained distances (i.e., visual acuity improvements were significant for both near and distance visual acuity) and even stereopsis. In addition, we now show that the training effects translate into improvements of daily life functioning (as assessed with a functional vision questionnaire), and that they are long lasting.

### Specificity and Transfer

Specificity gives insight into the processes underling experience-dependent plasticity but can be an obstacle in the development of efficient rehabilitation protocols. Here we find that promoting conditions for generalization (see section “Introduction”) results in broad transfer to task relevant conditions (reading) and task irrelevant conditions (stereopsis), culminating in significant improvements of daily life visual functioning in children and adolescents with IN. Our VPL task is a letter discrimination training in which stimuli unpredictably appeared on the left or right side of a central probe and subjects had to direct their attention and gaze to an eccentric target, addressing a wide range of visual functions (i.e., processing speed, fine discrimination, spatial attention and accurate gaze direction). Targeting multiple features heightens the change for broad transfer (Sasaki et al., 2010) and sensorimotor areas might have played a key role (Law and Gold, 2008). The long-lasting nature of these effects supports the notion that the improvements are mediated by VPL, but some caution is called for. By embedding the training in an oculomotor task, they may have learned to better focus and/or redirect their attention. In this way, children might improve their performance on other tasks even though their saccadic reaction times did not change (not shown, but see Huurneman et al., 2016a). We can rule out the possibility that the generalization resulted from enhanced contrast sensitivity. These finding suggests that our training primarily influenced higher-level visual processing.

A limitation of the current study is that it did not include a control group. All subjects received the same treatment and, therefore, we could not test whether the eye movement component of the learning task did or did not contribute to (the generalization of) the learning effects. We did collect test-retest data for the single letter and crowded letter discrimination paradigm in a previous study and we have published information about test-retest visual acuity data (Huurneman et al., 2016c). These findings show that test-retest learning effects are negligible compared to the reported effects of training. Moreover, in a recent study we showed that natural maturation effects on visual acuity over a time period of 8–14 months are small (<0.05 logMAR) in a sample of younger children (age range 4–8 years, Huurneman et al., 2020). In the current study, we also measured spatial and temporal aspects of reading performance, which are likely to be more heavily influenced by natural development occurring between 7 and 18 years of age than visual acuity measures. Our study results show that improvements in reading speed were twice as large between T0 and T1 (i.e., after 5 months of training) compared to the improvement observed between T1 and T2 (i.e., after 6 months without training), indicating that only part of the improvements between T0 and T1 can be attributed to natural development.

### Benefit of Prolonged Training

Improvements in crowded distance acuity and reading speed were larger than those observed in our previous project (see Supplementary Table 4 for a comparison of the training effects that were measured in both studies). The average improvement in crowded DVA measured with a standard vision chart after 10 training sessions was 0.11 ± 0.02 logMAR (Huurneman et al., 2016c) (pooled for the two diagnostic groups), while in the current project the effect after an average number of 37 training sessions equaled 0.18 ± 0.02 logMAR. In addition, maximum reading speed improved significantly in present study (15 ± 2 wpm), but not in the first (0 ± 1 wpm). Average learning effects on single letter discrimination and crowding extent, on the other hand, were remarkably similar to those reported earlier (Huurneman et al., 2017). The small impact of training on single letter acuity was expected, since there were fewer uncrowded than crowded letter training sessions. Note, however, that these comparisons are only group-level comparisons and that our previous study evaluated neither the level of retention nor the effects of training on daily life visual functioning.

Analysis of the individual learning curves indicated that the majority of our participants (26/36) needed more than 20 training sessions (10 uncrowded and 10 crowded training sessions) to reach a stable learning plateau (Figure 9B). The average learning curve indicates that 26 crowded training sessions were needed to reach plateau performance. A potential danger of prolonged training is that overtraining might resulting in a lack of generalization (Jeter et al., 2010). Here, we found no negative effects of prolonged training. Transfer of learning to untrained distances, reading performance and stereopsis was as good or better (see larger improvements in distance crowded visual acuity as measured with clinical charts) than in our previous study (Huurneman et al., 2016c).

### Other Factors Influencing Learning

Diagnosis and age did not correlate with the number of training sessions needed to reach plateau or the steepness of the learning curve. There was also no statistically significant relation between baseline crowded DVA and training induced changes in crowded DVA (*r* = –0.10, *p* = 0.548). However, poorer baseline performance on the crowded letter task was associated with the performance improvements on the crowded letter task (*r* = –0.37, *p* = 0.029). This finding is consistent with findings in adults with amblyopia (Levi and Li, 2009) and adults with normal vision (Yehezkel et al., 2016). A recent review indicates that age, treatment type (dichoptic treatment, perceptual learning, and videogames), amblyopia type and training duration were not predictive of VA improvements (Tsirlin et al., 2015). The finding that age does not affect treatment outcome in VPL is in contrast with amblyopia patching studies in which age does predict improvement (Wu and Hunter, 2006). Vision improvements induced by VPL and patching may result from different learning mechanisms with VPL depending less on retinotopic early visual cortex plasticity and more on non-retinotopic higher brain areas involved in attention engagement and decision making (Xiao et al., 2008). However, the locus of VPL is not a fixed concept and seems to depend on training factors (Maniglia and Seitz, 2018). There is evidence that older adults (aged 60-86 years) benefit as much from VPL as young adults (aged 18-35 years) (Andersen et al., 2010; Li et al., 2017). In sum, VPL appears to be an effective strategy to improve vision across life span, but it is still difficult to predict training outcome based on individual characteristics (e.g., diagnosis, treatment type or training duration).

### Differences in Learning Effects Between Diagnostic Groups

Children with idiopathic IN showed significantly larger improvements than children with albinism on measures that capture crowding: (1) crowded DVA, and (2) distance crowding intensity. These new findings corroborate our idea that albinism limits perceptual learning by more fundamental abnormalities of the visual system compared with idiopathic IN (Huurneman et al., 2017). Many hereditary forms of IN (e.g., the x-linked forms isolated to the FRMD7 gene, Thomas et al., 2008) do not have a detectable ocular defects and there is lack of consensus about the neural mechanism underlying IN (Abadi, 2002; Harris and Berry, 2006; Gottlob and Proudlock, 2014). There is also lack of consensus about the mechanism underlying amblyopia (Maurer and Mc, 2018; Kiorpes, 2019). Both IN and amblyopia may be regarded as neurodevelopmental disorders of the visual system (Harris and Berry, 2006; Maurer and Mc, 2018) and both appear to be particularly receptive to VPL. By contrast, effects of VPL in individuals with ocular impairment seem to be smaller and less stable. For example, in patients with a retinal defect (Stargardt disease) ten sessions of VPL yielded an initial improvement of reading speed and VA, but these improvements were no longer visible 6 months later (Sasso et al., 2019). However, there is evidence of long-lasting PL effect in age-related macular degeneration, which is similar to Stargardt’s disease (Maniglia et al., 2016). We think that there may be two reasons why individuals with visual developmental disorders tend to benefit more from VPL than those with ocular deficits. First, the adaptive abilities of the eye may be more limited than the adaptive abilities of the human brain (plasticity principle). Second, the quality of visual inputs to the brain will always be limited by the optical and sensory properties of the eye (garbage in - garbage out principle).

### Retention of Learning Effects

Nearly all training effects were stable over the 6 months follow-up period. These findings extend previous findings on the long-term effects of perceptual learning (Hussain et al., 2012; Huurneman et al., 2020). We cannot completely rule out that some of the improvements were also due to natural maturation. The mean age of our participants was 10 years and 11 months, and at this age, visual functions (Jeon et al., 2010), and reading skills are still under development (Kwon et al., 2007). However, improvements in maximum reading speed occurring over the 5-months training period were two times larger (i.e., 16 wpm) than the improvements occurring in the 6-months without training between the first and second post-test (i.e., 8 wpm). To our knowledge, there are no studies reporting the changes in children’s reading speed as a function of age for the Radner test. There is a study which evaluated changes in reading skills as a function of age with the MNREAD test in normally sighted individuals aged 8-81 years (Calabrese et al., 2016). This study showed that in 8 to 16-year-old children, the average reading speed increases linearly with age by a factor of 8.3 wpm/year and that the average reading acuity improves by 0.01 logMAR/year in this age range. By comparison, the average improvement in reading speed that we found in children with IN after 20 weeks of training was almost two times larger than the average improvement in reading speed one might expect for a normally sighted child over a time period of 1 year and three times as large over the total duration of our study. Taken together, these findings indicate that training had a positive impact on the development of reading speed on top of natural maturation effects.

## Conclusion

Training with an engaging perceptual learning app at home elicits broad and long-lasting vision improvements in youth with infantile nystagmus. This finding establishes perceptual learning as a valid, non-invasive treatment option to ameliorate the lifelong visual impairments associated with IN.

## Data Availability Statement

The raw data supporting the conclusions of this article will be made available by the authors, without undue reservation.

## Ethics Statement

The study was conducted according to the principles of the Declaration of Helsinki and approved by the local ethics committee (CMO Arnhem-Nijmegen, protocol ID NL61860.019.17, Netherlands Trial Register, trial number NL6711). Written informed consent to participate in this study was provided by the participants or the participants’ legal guardian/next of kin.

## Author Contributions

BH was involved with data collection and wrote the first version of the manuscript. Both authors were involved in data analysis, contributed to the interpretation of data, reviewed the manuscript, approved the submitted version, and designed the work.

## Conflict of Interest

The authors declare that the research was conducted in the absence of any commercial or financial relationships that could be construed as a potential conflict of interest.

## Publisher’s Note

All claims expressed in this article are solely those of the authors and do not necessarily represent those of their affiliated organizations, or those of the publisher, the editors and the reviewers. Any product that may be evaluated in this article, or claim that may be made by its manufacturer, is not guaranteed or endorsed by the publisher.
