# Supplementary figures and images for: Broad and Long-Lasting Vision Improvements in Youth With Infantile Nystagmus After Home Training With a Perceptual Learning App
Source: Front Neurosci. 2021 Aug 19;15:651205. doi: 10.3389/fnins.2021.651205 (PMC8417383; doi:10.3389/fnins.2021.651205)

# Group data

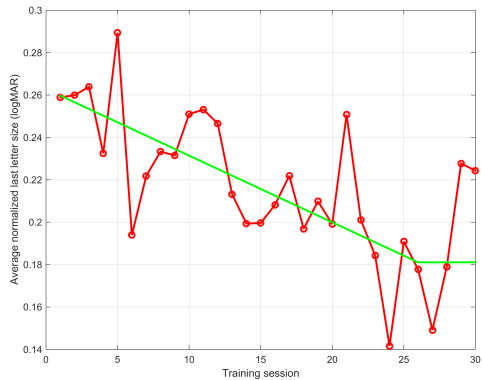

# Individual data

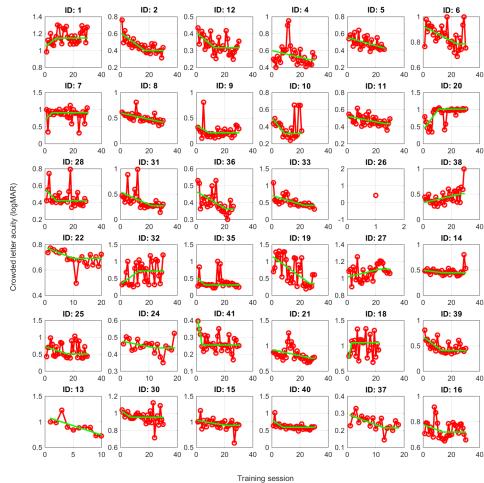

Supplement: Supplementary file 1 [file Data_Sheet_1.PDF]
