# Supplementary material for: Broad and Long-Lasting Vision Improvements in Youth With Infantile Nystagmus After Home Training With a Perceptual Learning App
Source: Front Neurosci. 2021 Aug 19;15:651205. doi: 10.3389/fnins.2021.651205 (PMC8417383; doi:10.3389/fnins.2021.651205)
Supplement: Supplementary file 2 [file Table_1.docx]

**Supplementary Table 1.** Clinical characteristics of the included children. Listed are age at inclusion [years;months], diagnosis, sex, crowded visual acuities [decimal acuity], stereopsis [sec arc], refractive corrections and number of completed training sessions.

| **#ID** | **Age** | **Diagnosis** | **Sex** | **DVA** | | | **NVA** | **Stereopsis** | **Correction** | # completed training sessions |
| --- | --- | --- | --- | --- | --- | --- | --- | --- | --- | --- |
|  |  |  |  | **OD** | **OS** | **ODS** |  |  |  |  |
| 1 | 10;6 | Oculo-cutaneous albinism | M | 0.13 | 0.10 | 0.16 | 0.16 | 800 | RE: S: +6.75 C: -0.50 ax: 20  LE: S: +7.25 C: -2.50 ax: 90 | 40 |
| 2 | 7;2 | IIN | M | 0.16 | 0.16 | 0.16 | 0.25 | 200 | RE: S: +5.00 C:-1.25 ax: 175  LE: S: +5.00 C:-0.75 ax: 160 | 40 |
| 4 | 13;2 | IIN | M | 0.20 | 0.20 | 0.32 | 0.32 | 800 | RE: S:-2.75 C:-2.75 ax: 80  LE: S:-1.75 C:-2.25 ax: 20 | 40 |
| 5 | 16;2 | Ocular albinism | M | 0.32 | 0.20 | 0.32 | 0.32 | 140 | RE: S:+4.00 C-3.00 ax: 95  LE: S: +4.00 C:-2.75 ax: 105 | 36 |
| 6 | 9;4 | Oculo-cutaneous albinism | F | 0.13 | 0.13 | 0.13 | 0.08 | 3,552 | RE: S: +7.25 C:-2.75 ax: 160  LE: S: +7.50 C:-3.75 ax: 20 | 40 |
| 7 | 8;3 | Ocular albinism | M | 0.13 | 0.16 | 0.16 | 0.16 | 400 | No correction | 40 |
| 8 | 14;8 | IIN | M | 0.32 | 0.32 | 0.40 | 0.25 | 400 | RE: S: -0.25 C:-2.00 ax: 160  LE: S: +0.50 C:-3.00 ax: 5 | 40 |
| 9 | 9;1 | IIN | M | 0.60 | 0.32 | 0.60 | 0.80 | 40 | No correction | 40 |
| 10 | 9;6 | IIN | M | 0.32 | 0.32 | 0.32 | 0.40 | 80 | RE: +1.75 C:-1.25 ax: 50  LE: +1.25 C:-1.00 ax: 110 | 31 |
| 11 | 10;9 | Oculo-cutaneous albinism | M | 0.20 | 0.32 | 0.25 | 0.25 | 800 | No correction | 40 |
| 12 | 12;6 | IIN | M | 0.25 | 0.25 | 0.25 | 0.40 | 100 | RE: -2.75 C:-1.00 ax: 175  LE: -2.75 C:-2.00 ax:170 | 40 |
| 13 | 7;0 | Oculo-cutaneous albinism | F | 0.10 | 0.08 | 0.13 | 0.13 | 800 | RE:+3.50 C:-1.25 ax: 10  LE:+3.50 C:-1.00 ax: 10 | 20 |
| 14 | 9;6 | Ocular albinism | F | 0.20 | 0.16 | 0.25 | 0.32 | 200 | No correction | 40 |
| 15 | 8;11 | Ocular albinism | F | 0.08 | 0.10 | 0.10 | 0.10 | 800 | RE:-4.25 C:-3.75 ax: 10 LE: -4.25 C:-4.50 ax: 170 | 40 |
| 16 | 16;10 | Ocular albinism | M | 0.20 | 0.20 | 0.25 | 0.20 | 800 | No correction | 40 |
| 18 | 8;9 | Oculo-cutaneous albinism | M | 0.13 | 0.13 | 0.16 | 0.20 | 800 | RE:-6.00 C:-4.50 ax: 175  LE:-5.75 C:-5.25 ax: 5 | 32 |
| 19 | 7;11 | IIN | F | 0.25 | 0.16 | 0.32 | 0.32 | 400 | RE:+6.25 C:-1.50 ax: 20  LE: +6.75 C:-2.00 ax: 160 | 40 |
| 20 | 7;0 | IIN | M | 0.16 | 0.20 | 0.25 | 0.32 | 400 | No correction | 40 |
| 21 | 17;11 | Ocular albinism | M | 0.10 | 0.13 | 0.13 | 0.10 | 3,552 | No correction | 40 |
| 22 | 16;5 | Oculo-cutaneous albinism | F | 0.08 | 0.16 | 0.16 | 0.13 | 800 | No correction | 30 |
| 24 | 13;2 | IIN | M | 0.25 | 0.25 | 0.32 | 0.32 | 140 | RE: -1.25 C:-0.75 ax:165  LE: -1.25 C:-1.00 ax: 160 | 29 |
| 25 | 11;1 | IIN | M | 0.16 | 0.25 | 0.25 | 0.25 | 800 | RE: +2.50 C:-2.50 ax: 150  LE: +1.25 C:-0.50 ax: 15 | 40 |
| 26 | 14;8 | Ocular albinism | M | 0.32 | 0.32 | 0.40 | 0.32 | 50 | No correction | 11 |
| 27 | 9;7 | Oculo-cutaneous albinism | M | 0.08 | 0.10 | 0.08 | 0.063 | 3,552 | RE:+5.00 C-4.00 ax:155  LE:+5.25 C:-4.25 ax: 25 | 40 |
| 28 | 8;2 | IIN | M | 0.16 | 0.25 | 0.16 | 0.32 | 140 | RE:-6.00 C:-4.50 ax:175  LE:-5.75 C:-5.25 ax: 5 | 39 |
| 30 | 11;11 | Oculo-cutaneous albinism | M | 0.10 | 0.13 | 0.10 | 0.08 | 800 | RE: +4.25 -1.75 ax: 25  LE: +4.50 -2.00 ax: 160 | 40 |
| 31 | 8;3 | Oculo-cutaneous albinism | M | 0.32 | 0.20 | 0.50 | 0.40 | 200 | RE: +2.00 C:-1.50 ax: 15  LE: +2.50 C:-1.50 ax: 25 | 40 |
| 32 | 15;0 | IIN | M | 0.32 | 0.25 | 0.40 | 0.40 | 3,552 | RE: -1.25 C: nil  LE: -1.25 C: nil | 40 |
| 33 | 8;0 | IIN | F | 0.32 | 0.25 | 0.40 | 0.50 | 140 | RE: +2.25 -0.25 ax: 30  LE: +2.00 -1.00 ax: 170 | 40 |
| 35 | 10;5 | Oculo-cutaneous albinism | M | 0.40 | 0.25 | 0.40 | 0.40 | 140 | No correction | 40 |
| 36 | 10;1 | IIN | F | 0.32 | 0.25 | 0.25 | 0.32 | 140 | RE: -0.75 C:-2.50 ax: 150  LE: -0.75 C:-2.50 ax: 15 | 36 |
| 37 | 10;7 | IIN | M | 0.32 | 0.25 | 0.32 | 0.40 | 60 | RE:-0.25 C:-3.00 ax: 5  LE:-0.25 C:-3.25 ax: 10 | 28 |
| 38 | 9;11 | Ocular albinism | F | 0.32 | 0.32 | 0.40 | 0.50 | 140 | No correction | 39 |
| 39 | 8;1 | IIN | M | 0.20 | 0.32 | 0.32 | 0.40 | 3,552 | RE:+1.75 C:-1.25 ax: 5  LE:+1.25 C:-1.00 ax: 170 | 40 |
| 40 | 13;1 | Oculo-cutaneous albinism | M | 0.20 | 0.16 | 0.16 | 0.20 | 800 | RE:+3.50 C:-3.50 ax: 100  LE:+3.75 C:-3.00 ax: 35 | 40 |
| 41 | 10;11 | IIN | F | 0.32 | 0.32 | 0.40 | 0.50 | 80 | No correction | 40 |

Abbreviations: IIN, Idiopathic Infantile Nystagmus; DVA, Distance Visual Acuity; NVA, Near Visual Acuity; RE, Right eye; LE, Left eye; S, Spherical correction; C, Cylinder correction, ax, axis orientation of astigmatism.
