# Supplementary material for: Broad and Long-Lasting Vision Improvements in Youth With Infantile Nystagmus After Home Training With a Perceptual Learning App
Source: Front Neurosci. 2021 Aug 19;15:651205. doi: 10.3389/fnins.2021.651205 (PMC8417383; doi:10.3389/fnins.2021.651205)
Supplement: Supplementary file 3 [file Table_2.docx]

**Supplementary Table 2.** Baseline visual acuities, stereopsis and task-specific measures of children with albinism (Alb) and idiopathic infantile nystagmus (IIN) (mean ± SD).

|  | **Alb.**  **(n=19)** | **IIN**  **(n=17)** | ***t*-statistic** | ***p*-value** | **Direction of the effect** |
| --- | --- | --- | --- | --- | --- |
| **Age**  **[months]** | 138 ± 40 | 124 ± 31 | 1.12 | 0.271 | n.s. |
| **Uncrowded DVA [logMAR]** | 0.56±0.27 | 0.29±0.14 | 3.60 | <0.001 | Alb > IIN |
| **Crowded DVA [logMAR]** | 0.71±0.23 | 0.52±0.14 | 2.97 | 0.005 | Alb > IIN |
| **Uncrowded NVA [logMAR]** | 0.53±0.27 | 0.21±0.11 | 4.49 | <0.001 | Alb > IIN |
| **Crowded NVA [logMAR]** | 0.73±0.27 | 0.44±0.13 | 4.21 | <0.001 | Alb > IIN |
| **Distance Crowding Intensity**  **[logMAR]** | 0.16±0.09 | 0.22±0.12 | -1.96 | 0.058 | n.s. |
| **Near Crowding Intensity**  **[logMAR]** | 0.21±0.09 | 0.23±0.09 | -0.45 | 0.656 | n.s. |
| **Stereopsis**  **[log_10_(sec arc)]** | 2.71±0.48 | 2.37±0.51 | 2.10 | 0.044 | Alb > IIN |
| **Uncrowded VA  single letter task [logMAR]** | 0.54±0.25 | 0.27±0.22 | 3.40 | 0.002 | Alb > IIN |
| **Crowding Extent [logMAR]** | 1.13±0.31 | 0.86±0.29 | 2.71 | 0.010 | Alb > IIN |
