# Supplementary material for: Broad and Long-Lasting Vision Improvements in Youth With Infantile Nystagmus After Home Training With a Perceptual Learning App
Source: Front Neurosci. 2021 Aug 19;15:651205. doi: 10.3389/fnins.2021.651205 (PMC8417383; doi:10.3389/fnins.2021.651205)
Supplement: Supplementary file 4 [file Table_3.docx]

**Supplementary Table 3.** Baseline reading measures, contrast sensitivity measures and FVQ-scores of children with albinism (Alb) and idiopathic infantile nystagmus (IIN) (mean ± SD).

|  | **Alb.**  **(n=19)** | **IIN**  **(n=17)** | ***t*-statistic** | ***p*-value** | **Direction** |
| --- | --- | --- | --- | --- | --- |
| **Reading acuity [logMAR]** | 0.78±0.25 | 0.46±0.25 | -4.73 | <0.001 | Alb>IIN |
| **Critical Print Size [logMAR]** | 1.09±0.26 | 0.81±0.18 | -3.64 | <0.001 | Alb>IIN |
| **Acuity Reserve [logMAR]** | 0.32±0.14 | 0.35±0.16 | 0.74 | 0.466 | n.s. |
| **Maximum Reading Speed [wpm]** | 113±37 | 100±34 | -1.11 | 0.273 | n.s. |
| **Weber contrast sensitivity level 1** | 59.1±23.5 | 71.1±30.6 | 1.06 | 0.300 | n.s. |
| **Weber contrast sensitivity level 2** | 44.9±10.1 | 52.4±29.6 | 0.88 | 0.386 | n.s. |
| **Weber contrast sensitivity level 3** | 27.4±8.1 | 36.1±11.2 | 2.17 | 0.042 | IIIN>Alb |
| **Weber contrast sensitivity level 4** | 12.2±6.0 | 16.3±12.6 | 1.05 | 0.304 | n.s. |
| **Weber contrast sensitivity level 5** | 3.5±2.6 | 5.5±5.4 | 1.20 | 0.243 | n.s. |
| **FVQ-home** | 7.5±3.7 | 7.6±2.9 | 0.11 | 0.915 | n.s. |
| **FVQ-school** | 17.2±7.2 | 16.5±6.1 | -0.33 | 0.742 | n.s. |
| **FVQ-mobility** | 6.7±4.5 | 7.5±3.3 | 0.69 | 0.497 | n.s. |
| **FVQ-leisure** | 4.8±2.9 | 5.1±2.9 | 0.28 | 0.778 | n.s. |
