# Supplementary material for: Broad and Long-Lasting Vision Improvements in Youth With Infantile Nystagmus After Home Training With a Perceptual Learning App
Source: Front Neurosci. 2021 Aug 19;15:651205. doi: 10.3389/fnins.2021.651205 (PMC8417383; doi:10.3389/fnins.2021.651205)
Supplement: Supplementary file 5 [file Table_4.docx]

**Supplementary Table 4.** Comparison of training effects between studies (mean ± standard error of the mean). In the first study (Huurneman et al. IOVS 2016;57(10):4216-4246) there were two training groups: an uncrowded and crowded training group. The current study evaluates prolonged training effects and all subjects received the same training (i.e. first 10 uncrowded and then 30 crowded sessions).

|  | **After 10 training sessions** | **After 37 training sessions** | **Group**  **Difference** |
| --- | --- | --- | --- |
| **Clinical charts** | | | |
| **Δ Uncrowded DVA [logMAR]** | 0.10 ± 0.02 | 0.15 ± 0.02 | 0.05 ± 0.03 (n.s.) |
| **Δ Crowded DVA [logMAR]** | 0.11 ± 0.02 | IIN: 0.24 ± 0.02  Alb: 0.13 ± 0.02 | 0.13 ± 0.03***  0.02 ± 0.03 (n.s.) |
| **Δ Uncrowded NVA [logMAR]** | 0.07 ± 0.02 | 0.09 ± 0.02 | 0.02 ± 0.03 (n.s.) |
| **Δ Crowded NVA [logMAR]** | Uncr. tr.: 0.05 ± 0.03  Cr. tr: 0.15 ± 0.02 | 0.10 ± 0.02 | 0.05 ± 0.04 (n.s.)  -0.05 ± 0.03 (n.s.) |
| **Δ Distance Crowding Intensity**  **[logMAR]** | 0.02 ± 0.02 | IIN: 0.08 ± 0.03  Alb: -0.01 ± 0.03 | 0.06 ± 0.04 (n.s.)  -0.03 ± 0.04 (n.s.) |
| **Δ Near Crowding Intensity**  **[logMAR]** | Uncr. tr: -0.02 ± 0.03  Cr. tr. : 0.08 ± 0.03 | 0.01 ± 0.02 | 0.03 ± 0.04 (n.s.)  -0.07 ± 0.04 (n.s.) |
| **Δ Stereopsis**  **[log_10_(sec arc)]** | 0.35 ± 0.11 | 0.19 ± 0.06 | -0.14 ± 0.13 (n.s.) |
| **Training task** | | | |
| **Δ Uncrowded VA  single letter task [logMAR]** | 0.085 ± 0.014 | 0.064 ± 0.017 | -0.02 ± 0.02 (n.s.) |
| **Δ Crowding Extent [logMAR]** | 0.25 ± 0.04 | 0.29 ± 0.03 | 0.04 ± 0.05 (n.s.) |
| **Reading performance** | | | |
| **Δ Reading Acuity [logMAR]** | 0.12 ± 0.02 | 0.07 ± 0.02 | -0.05 ± 0.03 (n.s.) |
| **Δ Critical Print Size [logMAR]** | 0.11 ± 0.04 | 0.07 ± 0.04 | -0.04 ± 0.06 (n.s.) |
| **Δ Maximum Reading Speed [wpm]** | 0 ± 1 wpm | 15 ± 2 wpm | 15 ± 2*** |
| **Δ Acuity Reserve [logMAR]** | -0.02 ± 0.04 | 0.00 ± 0.03 | 0.02 ± 0.05 (n.s.) |

n.s. = not significant, *** *p* < 0.001, Uncr. = uncrowded training, Cr. = crowded training
